# The Risk of Postpartum Hemorrhage Following Prior Prelabor Cesarean Delivery Stratified by Abnormal Placentation: A Multicenter Historical Cohort Study

**DOI:** 10.3389/fmed.2021.745080

**Published:** 2021-10-11

**Authors:** Bi Shilei, Zhang Lizi, Li Yulian, Liang Yingyu, Huang Lijun, Huang Minshan, Huang Baoying, Jia Jinping, Cao Yinli, Wang Shaoshuai, Xu Xiaoyan, Feng Ling, Zhao Yangyu, Zhao Xianlan, Zhu Qiying, Qi Hongbo, Wen Suiwen, Zhang Lanzhen, Li Hongtian, Chen Jingsi, Wang Zhijian, Du Lili, Chen Dunjin

**Affiliations:** ^1^Key Laboratory for Major Obstetric Diseases of Guangdong Province, Department of Obstetrics and Gynecology, The Third Affiliated Hospital of Guangzhou Medical University, Guangzhou, China; ^2^Department of Obstetrics and Gynecology, Nanfang Hospital, Southern Medical University, Guangzhou, China; ^3^Department of Obstetrics and Gynecology, Guangzhou Huadu District Maternal and Child Health Hospital, Guangzhou, China; ^4^Department of Obstetrics and Gynecology, Northwest Women's and Children's Hospital, Xian, China; ^5^Department of Obstetrics and Gynecology, Tongji Hospital, Tongji Medical College, Huazhong University of Science and Technology, Wuhan, China; ^6^Department of Obstetrics and Gynecology, Peking University Third Hospital, Beijing, China; ^7^Department of Obstetrics and Gynecology, The First Affiliated Hospital of Zhengzhou University, Zhengzhou, China; ^8^Department of Obstetrics and Gynecology, The First Affiliated Hospital of Xinjiang Medical University, Urumqi, China; ^9^Department of Obstetrics and Gynecology, The First Affiliated Hospital of Chongqing Medical University, Chongqing, China; ^10^Department of Obstetrics and Gynecology, The Sixth Affiliated Hospital of Guangzhou Medical University, Qingyuan People's Hospital, Guangzhou, China; ^11^Department of Obstetrics and Gynecology, The Second Affiliated Hospital of Guangzhou Medical University, Guangzhou, China; ^12^National Health Commission Key Laboratory of Reproductive Health, Institute of Reproductive and Child Health, Peking University Health Science Center, Beijing, China; ^13^Key Laboratory of Reproduction and Genetics of Guangdong Higher Education Institute, Guangzhou, China

**Keywords:** postpartum hemorrhage (PPH), cesarean delivery (CD), placenta previa, placenta accreta spectrum (PAS), trial of labor (TOL)

## Abstract

**Background:** Prior prelabor cesarean delivery (CD) was associated with an increase in the risk of placenta previa (PP) in a second delivery, whether it may impact postpartum hemorrhage (PPH) independent of abnormal placentation. This study aimed to assess the risk of PPH stratified by abnormal placentation following a first CD before the onset of labor (prelabor) or intrapartum CD.

**Methods:** This multicenter, historical cohort study involved singleton, pregnant women at 28 weeks of gestation or greater with a CD history between January 2017 and December 2017 in 11 public tertiary hospitals within 7 provinces of China. PPH was analyzed in the subsequent pregnancy between women with prior prelabor CD and women with intrapartum CD. Furthermore, PPH was analyzed in pregnant women stratified by complications with PP alone [without placenta accreta spectrum (PAS) disorders], complications with PP and PAS, complications with PAS alone (without PP), and normal placentation. We performed multivariate logistic regression to calculate adjusted odds ratios (aOR) and 95% CI controlling for predefined covariates.

**Results:** Out of 10,833 pregnant women, 1,197 (11%) women had a history of intrapartum CD and 9,636 (89%) women had a history of prelabor CD. Prior prelabor CD increased the risk of PP (aOR 1.91, 95% CI 1.40–2.60), PAS (aOR 1.68, 95% CI 1.11–2.24), and PPH (aOR 1.33, 95% CI 1.02–1.75) in a subsequent pregnancy. After stratification by complications with PP alone, PP and PAS, PAS alone, and normal placentation, prior prelabor CD only increased the risk of PPH (aOR 3.34, 95% CI 1.35–8.23) in a subsequent pregnancy complicated with PP and PAS.

**Conclusion:** Compared to intrapartum CD, prior prelabor CD increased the risk of PPH in a subsequent pregnancy only when complicated by PP and PAS.

## Introduction

Postpartum hemorrhage (PPH) is a major contributor to maternal morbidity and death (1) and it also exerts long-term overall impact on health of women (2). The prevalence of PPH progressively increased from 6.3% in 2000 to 8% in 2009 in Canada (3), and it reached a level of 25.7% in Africa (4). Abnormal placentation including placenta previa (PP) (abnormal location of the lower edge of the placenta) and placenta accreta spectrum (PAS) (abnormal adherence of the placenta at the endometrial–myometrial interface that leads to aberrant decidualization) is the most common cause of PPH (5). Abnormal placentation is associated with damage to the endometrial–myometrial interface of the uterine wall, mainly uterine scar secondary to cesarean delivery (CD) (6). CD is a lifesaving procedure for women with complications, but the dramatic rise in the CD rate worldwide has raised concerns about the adverse maternal and neonatal outcomes and also the long-term consequences (7–9). It is, thus, important to prevent the overuse of CD, especially primary CD. Primary CD is classified as intrapartum CD and prior to the onset of labor (prelabor) according to the time of its execution. It is noteworthy that some investigators have reported that the long-term effects of these two types of CD are different (10–12).

Kamel et al. found that the location of the uterine incision differed between intrapartum CD and prelabor CD and that the latter increased the risk of a scar-niche formation (12). Downes et al. reported that prior prelabor CD increased the risk of placenta previa in the second delivery compared with prior intrapartum CD (10). In the study by Kamara et al., women with prelabor CD were more likely to develop a placenta accreta in the subsequent pregnancy complicated with placenta previa, relative to those women with intrapartum CD (11). Similarly, prelabor CD may be an additional risk factor for PPH compared with intrapartum CD since the uterine repair might be different after the trial of labor, even though it is failed (13).

Very little is known regarding the impact of prior CD subtype on PPH in a subsequent pregnancy independent of abnormal placentation.

In this study, we investigated the association between PPH and the subtype of prior CD (prelabor vs. intrapartum) as stratified by abnormal placentation.

## Methods

**Figure 1:**
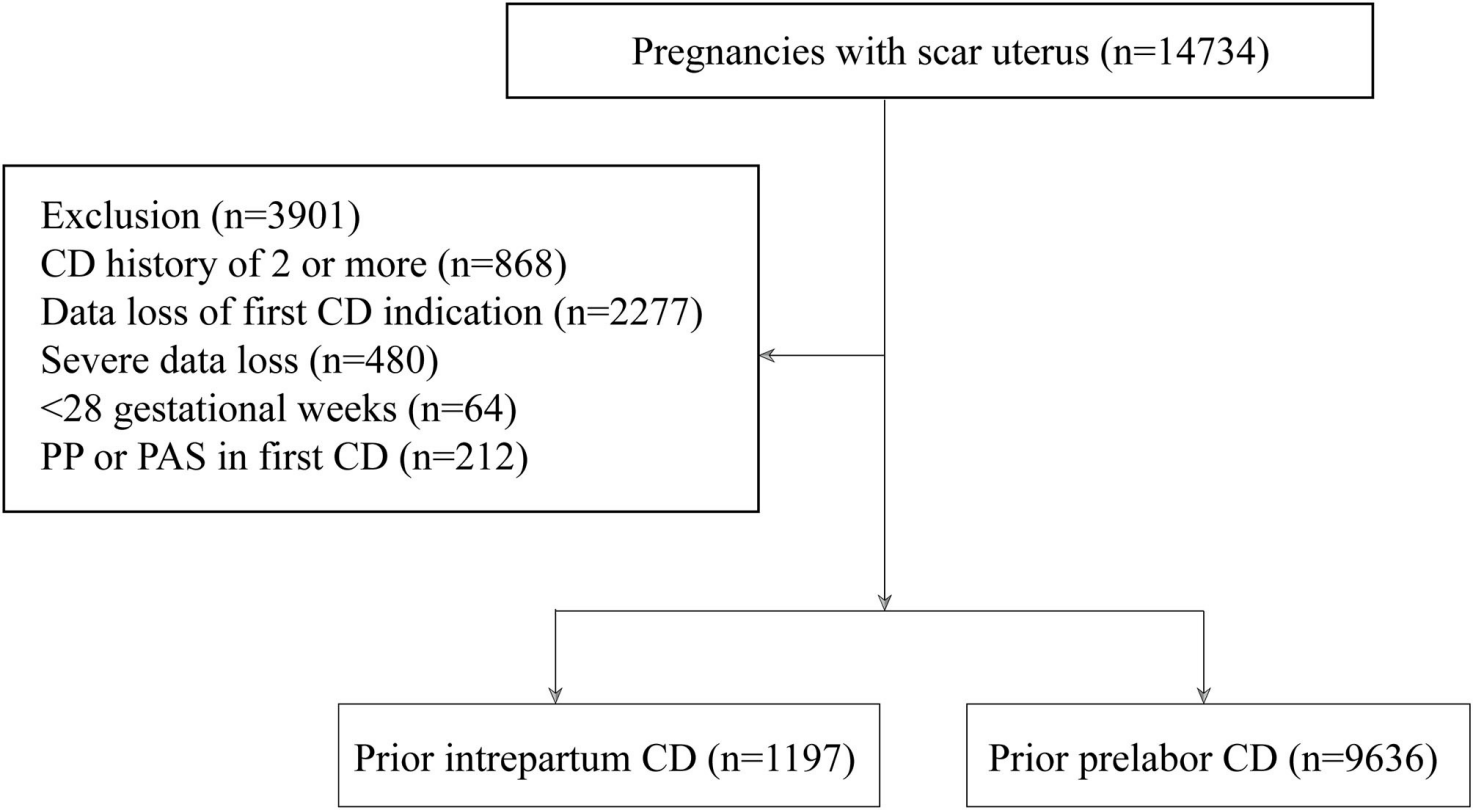
A flow diagram of the enrollment process of the patient.

This was a multicenter, historical, cross-sectional cohort study of pregnant women with a scarred uterus in 11 public tertiary hospitals covering 7 provinces, municipalities, and autonomous regions within China (Guangdong, Beijing, Xinjiang, Shanxi, Henan, Hubei, and Chongqing) from January 2017 to December 2017. Electronic medical records were used to identify women with a history of CD who delivered again and a review of the chart was performed to obtain further data. A history of two or more CDs, loss of CD indication records, a vertical uterine incision in a previous CD, severe data loss, and gestational age <28 weeks were excluded. Since most women with PP or PAS require CD before labor and prior PP and PAS increase the incidence of future PP and PAS (which are the risk factors for PPH in a subsequent pregnancy), (14) pregnant women with PP or PAS in a prior CD were excluded. First CD was categorized into the following: (1) CD before onset of labor (prelabor CD) or (2) CD after onset of labor (intrapartum CD). Figure 1 depicts a flow diagram of the patient enrollment process.

Maternal demographics of the index pregnancy included maternal age, prepregnancy body mass index (BMI), gestational weight gain, gravida, number of abortions, interpregnancy interval, race/ethnicity, use of assisted reproductive technology (ART), source of the women (referral or not), history of curettage, obstetric complications [premature rupture of membranes (PROM), hypertensive disorders, and diabetes mellitus (DM)], and mode of delivery. Maternal demographics of the preceding pregnancy included indication, history of CD, and complications.

Postpartum hemorrhage was analyzed in the subsequent pregnancy between women with prior prelabor CD and women with intrapartum CD. As PP and PAS were the highest risk factors for PPH, we further analyzed PPH in a subsequent pregnancy as stratified by PP, PAS, and normal placentation. Considering that PP itself is a risk factor for PAS disorders, we classified PP and PAS as PP alone (PP without PAS), PP complicated by PAS, and PAS alone (PAS without PP).

We defined PPH as a loss of ≥1,000 ml of blood after CD and ≥500 ml of blood after vaginal delivery (15). The blood loss was estimated principally through gravimetric measurement, i.e., we took the weight of blood-soaked items and subtracted the dry weight of the items to obtain blood loss volume. We also collected blood in the calibrated canisters through a negative pressure aspirator after the birth of the neonate, thus avoiding the problem of measuring non-blood fluids. PP was defined as a placenta lying directly over the internal cervical os and PAS disorders were distinguished using intraoperative findings or postoperative pathology.

## Statistical Analyses

We conducted statistical analyses using R software (version 3.6.1) and SPSS (version 25.0) for Windows. Quantitative data were examined for normal distribution using the Q–Q plot and the Kolmogorov–Smirnov test. Student's *t-*test and the Mann–Whitney *U-*test were used to compare continuous variables with normal and non-normal distributions between the two groups, respectively. Categorical variables were reported as a frequency (percentage) and the differences between the groups were compared using the Chi-squared or Fisher's exact probability test in cases of small numbers, as appropriate. We investigated the risk of PPH according to prior CD mode using multivariate logistic regression analysis adjusted for possible confounders—including age, weight gain, hypertensive disorders, and DM. We also calculated crude odds ratios (ORs) and adjusted odds ratios (aORs) along with their 95% CIs. Differences with *p* < 0.05 were considered to be statistically significant.

## Results

**Figure 2:**
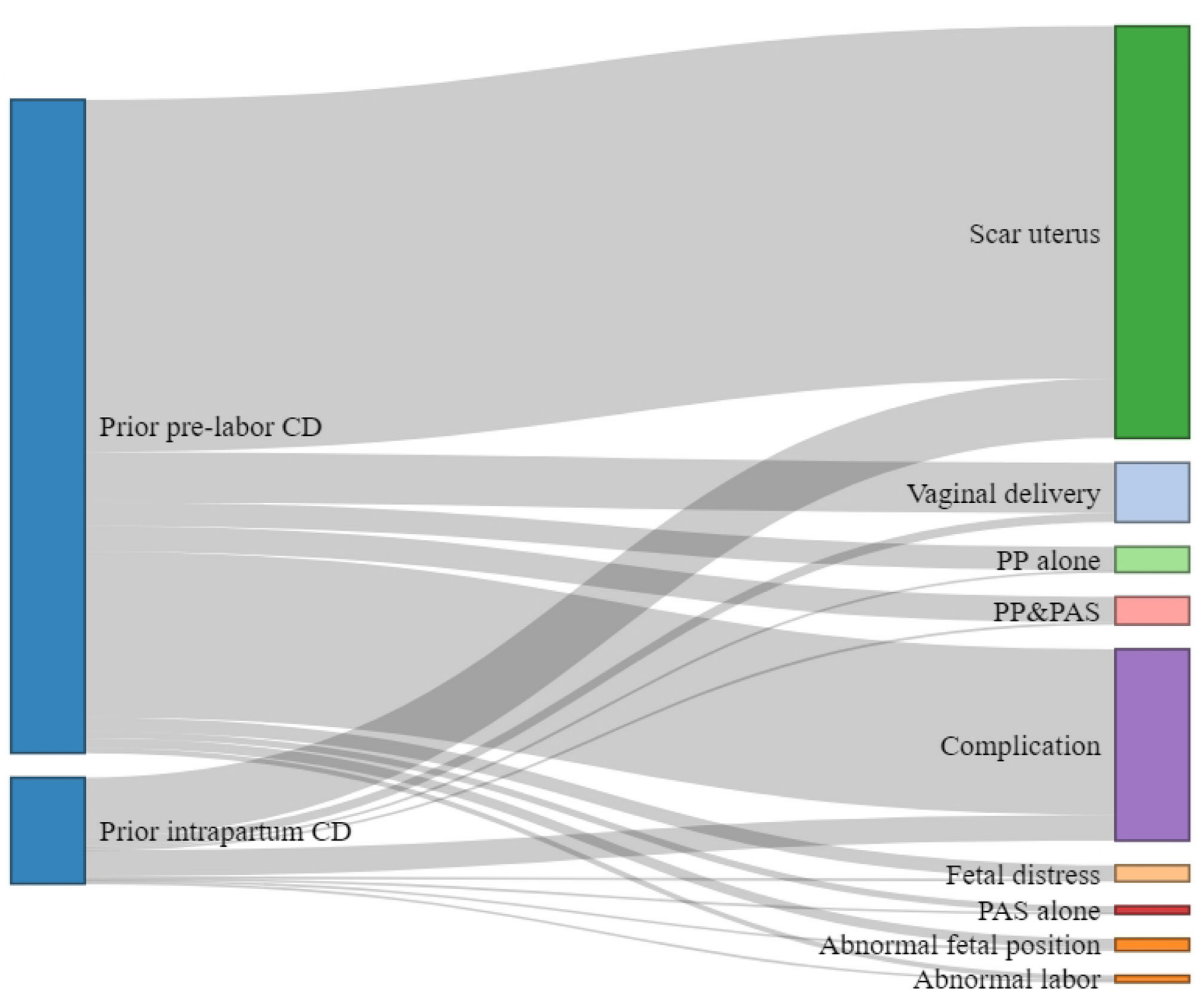
Vaginal deliveries and the indications of repeat cesarean deliveries (CDs) in a subsequent pregnancy in women with prior prelabor CD or intrapartum CD.

This study comprised 10,833 pregnant women with a history of CD. The majority of deliveries at the first pregnancy were prelabor CDs (89%, *n* = 9,636) followed by intrapartum CDs (11%, *n* = 1,197) (Figure 1). Figure 2 shows the vaginal deliveries and the indications of repeat CDs in a subsequent pregnancy in women with a prior prelabor CD or intrapartum CD. A total of 100 (8.4% of the total) pregnant women with prior intrapartum CD and 851 (8.8%) pregnant women with prior prelabor CD were delivered vaginally. The common indications for subsequent CD in women with prior intrapartum CD or prelabor CD were previous CD (56.3 vs. 57.9%), complications (24.5 vs. 24.0%), abnormal labor (1.6 vs. 0.6%), non-reassuring fetal trace (2.3 vs. 2.4%), PP alone (1.8 vs. 3.3%), PAS alone (1.0 vs. 1.0%), PP complicated by PAS (1.9 vs. 3.5%), and abnormal fetal position (2.3 vs. 1.4%).

Table 1 represents the demographics of a subsequent pregnancy. Pregnant women with prior prelabor CD were significantly more likely to be younger and manifest greater weight gain during pregnancy compared with women with prior intrapartum CD. Compared to women with prior intrapartum CD, women with prior prelabor CD exhibited a significantly higher prevalence of hypertensive disorders.

**Table 1:** Demographics of a subsequent pregnancy.

**Variables**	**Prior intrapartum CD**	**Prior prelabor CD**	***p***
	**(***n*** = 1,197)**	**(***n*** = 9,636)**	
Age	33.31 ± 4.21	33.08 ± 4.39	0.054
BMI	22.38 ± 3.15	22.56 ± 3.24	0.114
Gravida	2.90 ± 1.03	2.94 ± 1.06	0.308
Abortion	0.91 ± 1.03	0.97 ± 1.08	0.116
Curettage	8 (0.7)	117 (1.2)	0.095
Interpregnancy interval	82.16 ± 41.57	82.11 ± 42.29	0.207
Weight gain	13.07 ± 4.10	13.33 ± 3.90	0.006
Race/ethnicity			0.121
Han	1,170 (97.7)	9,478 (98.4)	
Others	27 (2.3)	158 (1.6)	
ART	23 (2.1)	255 (2.8)	0.147
Source			0.132
Hospital	1,073 (89.6)	8,495 (88.2)	
Referral	124 (10.4)	1,141 (11.8)	
Mode of delivery			0.173
Repeat CD	1,077 (90)	8,785 (91.2)	
VBAC	100 (8.4)	851 (8.8)	
PROM	130 (10.9)	974 (10.1)	0.417
Hypertension disorders	46 (3.8)	592 (6.1)	0.001
DM	258 (21.6)	1866 (19.4)	0.072

*Data are presented as mean ± SD or n (%). CD, cesarean delivery; BMI, body mass index; ART, assisted reproductive technology; VBAC, vaginal birth after a cesarean; PROM, premature rupture of membranes; DM, diabetes mellitus*.

Table 2 demonstrates the risks of PPH and blood transfusion that were observed among women with prior prelabor CD both before and after adjustments for maternal age, weight gain, hypertensive disorders, and DM. We also found that prior prelabor CD was the independent risk factor for antepartum hemorrhage, PP (including PP alone and PP complicated by PAS), and PAS (referring primarily to PAS complicated by PP and not PAS alone).

**Table 2:** Multivariate logistic regression analysis on all the subsequent pregnancies.

**Variables**	**Prior intrapartum CD (***n*** = 1,197)**	**Prior prelabor** **CD (***n*** = 9,636)**	**OR**	**95% CI**	**aOR**	**95% CI**	***p***
PPH	61 (5.1)	641 (6.7)	1.33	1.01–1.74	1.33	1.02–1.75	0.037
Blood transfusion	35 (3)	508 (5.3)	1.51	1.07–2.15	1.51	1.06–2.14	0.022
Antepartum hemorrhage	32 (2.7)	439 (4.6)	1.74	1.21–2.50	1.75	1.22–2.52	0.003
PP	45 (3.8)	655 (6.8)	1.87	1.37–2.54	1.91	1.40–2.60	0
PP alone	22 (1.8)	315 (3.3)	1.81	1.17–2.79	1.86	1.20–2.87	0.006
PAS	35 (2.9)	434 (4.5)	1.57	1.10–2.22	1.68	1.11–2.24	0.011
PAS and PP	23 (1.9)	340 (3.5)	1.87	1.22–2.86	1.89	1.23–2.90	0.004
PAS alone	12 (1)	94 (1)	1.03	0.56–1.88	1.04	0.57–1.90	0.901

*Data are presented as n (%). Values are presented as odds ratio (OR) (95% CI). Adjusted factors included maternal age, weight gain, hypertensive disorders, and diabetes mellitus. PPH, postpartum hemorrhage; CD, cesarean delivery; PP, placenta previa; PAS, placenta accreta spectrum; aOR, adjusted odds ratios*.

Prior prelabor CD only increased the risk of PPH and blood transfusions in women showing complications of PP with PAS in a subsequent pregnancy. However, the prior prelabor CD did not influence antepartum hemorrhage, PPH, or blood transfusions in women with normal placentation or complicated by PP alone or PAS alone (Table 3). The demographics of a subsequent pregnancy complicated by PP and PAS, PP alone, PAS alone, and normal placentation are illustrated in Supplementary Tables 1–4, respectively.

**Table 3:** Multivariate logistic regression analysis in pregnancies complicated by PP alone, PP and PAS, PAS alone, or normal placentation.

**Variables**		**Prior intrapartum CD**	**Prior prelabor CD**	**OR**	**95% CI**	**aOR**	**95% CI**	***p***
PP alone	Antepartum hemorrhage	4 (18.2)	97 (30.8)	2	0.66–6.07	2.05	0.67–6.30	0.21
(*n* = 337)	PPH	3 (13.6)	92 (29.2)	2.61	0.76–9.04	2.82	0.81–9.84	0.104
	Blood transfusion	2 (10)	62 (20)	2.25	0.51–9.96	2.33	0.52–10.44	0.268
PP and PAS	Antepartum hemorrhage	7 (30.4)	104 (30.6)	1.01	0.40–2.52	0.95	0.37–2.44	0.912
(*n* = 363)	PPH	8 (34.8)	211 (62.1)	3.07	1.27–7.44	3.34	1.35–8.23	0.009
	Blood transfusion	5 (21.7)	150 (44.1)	2.84	1.03–7.83	2.79	1.01–7.75	0.049
PAS alone	Antepartum hemorrhage	0 (0)	2 (2.1)	-		-		-
(*n* = 106)	PPH	2 (16.7)	21 (22.3)	1.44	0.29–7.08	1.79	0.33–9.60	0.498
	Blood transfusion	1 (8.3)	13 (13.8)	1.77	0.21–14.84	1.32	0.15–11.89	0.806
Normal placentation	Antepartum hemorrhage	21 (1.8)	236 (2.7)	1.45	0.93–2.28	1.45	0.92–2.27	0.109
(*n* = 10,027)	PPH	48 (4.2)	317 (3.6)	0.84	0.62–1.15	0.83	0.61–1.14	0.249
	Blood transfusion	27 (2.5)	203 (2.3)	0.94	0.63–1.42	0.93	0.62–1.40	0.722

*Data are presented as n (%). Values are presented as odds ratio (OR) (95% CI). Adjusted factors included maternal age, weight gain, hypertensive disorders, and diabetes mellitus. PPH, postpartum hemorrhage; CD, cesarean delivery; PP, placenta previa; PAS, placenta accreta spectrum; aOR, adjusted odds ratios*.

## Discussion

In this multicenter, historical, cross-sectional cohort study of pregnant women with a history of CD, we established that prior prelabor CD increased the risk of antepartum hemorrhage, PP, PAS (referring primarily to PAS complicated by PP and not PAS alone), PPH, and blood transfusion. After stratification by PP alone, PP complicated by PAS, and PAS alone, only prior prelabor CD increased the risk of PPH and blood transfusion in a subsequent pregnancy complicated by PP and PAS.

In the study by, Downes et al. (10) they used a history of PP as an adjusted factor to study the effect of prior delivery mode on subsequent PP. However, most of the women in their study who showed complications with a history of PP in their study chose CD directly without trial of labor, as did women with a history of PAS. In our study, we excluded pregnant women with a history of PP or PAS. In addition, as the prior CD itself is also a risk factor for PP compared with prior vaginal birth, we focused in a subsequent pregnancy on the outcome of women with a history of CD. A previous case-control study suggested that women with a primary elective CD without labor were likely to develop a placenta increta in a subsequent pregnancy with PP relative to those undergoing primary emergency CD with labor (11). Consistent with previous reports, we proved that prior prelabor CD increased the risk of PP complicated by PAS in a subsequent pregnancy. Furthermore, we found that prior prelabor did not increase the risk of PAS without the complication of PP.

Although antepartum hemorrhage is commonly caused by PP and placental abruption, placental abruption accounts for only a fraction of the cases of hemorrhage in pregnancy (16–18). The association between PP and PAS and the risk of PPH is well documented (19, 20) and the need for transfusion usually indicates greater severity of hemorrhage (18). In the present study, the increased risk of antepartum hemorrhage, PPH, and blood transfusion by prior prelabor might be due to prior prelabor CD, thus increasing the prevalence of PP and PAS. We then analyzed the association between subtypes of prior CD and PPH in a subsequent pregnancy complicated by PP and PAS, PP alone, PAS alone, or normal placentation, and, intriguingly, prior prelabor CD did not influence antepartum hemorrhage, PPH, or the need for blood transfusion in women with normal placentation, complicated by PP alone or by PAS alone. It has been suggested that the structure of the uterine segment, uterine activation, and immune function in women with intrapartum CD might reduce the damage and promote healing compared with prelabor CD (10). In addition, prelabor CD resulted in the scar being situated in the uterine cavity above the internal cervical os with more scar-niche formation. In contrast, intrapartum CD resulted in the scar being positioned in the uterine cavity at or below the internal cervical os with less scar-niche formation (12). All of the aforementioned factors may represent a plausible mechanism(s) for aberrant placentation in a subsequent pregnancy. PAS is often complicated with PP and its prevalence has been directly linked to a prior CD (21, 22). However, studies on the association between PAS alone and prior prelabor CD are rare and we speculate that pregnancies complicated by PAS alone in a subsequent pregnancy may be due to other reasons (e.g., curettage and hysteroscopy) instead of the prior CD.

Placenta accreta spectrum combined with PP is more likely to lead to PPH than PAS alone or PP alone (23). It has also been shown that bleeding often occurs from the deep area of the lower segment just after placental removal (24). One possible mechanism underlying the association between the subtype of prior CD and PPH in pregnancies complicated by PP and PAS may be due to the fact that prior prelabor CD was likely to predispose women to a uterine scar defect (12). Alternatively, an explanation might be related to the differences in the proportion of normally remodeled blood vessels (25). Therefore, to meet the local oxygen demands and metabolic requirements, the area and depth of the trophoblast invading the myometrium might increase (26), predisposing these women to severe bleeding and additional intrauterine intraoperative procedures (27).

## Strengths and Limitations

Our study exhibited several strengths. At first, this was a multicenter study that involved 11 public tertiary hospitals covering 7 provinces, municipalities, and autonomous regions within China. We, thus, avoided the selection bias inherent to a single-center study and the study would be more generalizable to heterogeneous populations. Second, this was the first study on the association between PPH in a subsequent pregnancy and subtype of prior CD independent of PAS and PP. However, our study possessed some limitations. This was a historical cohort study using a review of the chart and some data were not available in every hospital's dataset. We were unable to further classify PP (marginal, partial, or complete PP) and PAS (placenta accreta, placenta increta, and placenta percreta), which would have allowed us to better understand the relationship between the subtype of prior CD and abnormal placentation. After stratification by PP and PAS, the numbers of PPH cases were also relatively small in women with the prior prelabor CD or intrapartum CD. The intriguing results of this study require further examination in other population-based samples.

## Conclusion

In conclusion, the findings of the present study suggested that compared with intrapartum CD, prior prelabor CD increases PP, PAS, and PPH in a subsequent pregnancy; however, prelabor CD increases PPH only when complicated by PP and PAS. Though technological advances have made CD extremely safe (28), primary CD was not the first choice for pregnant women who did not accept intrapartum CD due to its potential long-term impact. We suggest that clinicians instill in pregnant women greater confidence and correct misunderstandings with respect to intrapartum CD in order to reduce the adverse maternal outcome in a subsequent pregnancy due to primary CD.

## Data Availability Statement

The raw data supporting the conclusions of this article will be made available by the authors, without undue reservation.

## Ethics Statement

This historical study was approved by the Medical Ethics Committee of Guangzhou Medical University with Medical Research No. 2016 (0406) approved on April 6, 2016. Written informed consent for participation was not required for this study in accordance with the national legislation and the institutional requirements.

## Author Contributions

BS and ZLi helped in conceptualization, methodology, software, validation, writing, review, and editing. LYu, CJ, and LYi contributed in investigation, review, and editing. HM, HL, and HB contributed in investigation and data collection. JJ, WSh, CY, WSu, XX, FL, ZX, ZY, ZQ, QH, ZLa, and LH contributed in investigation and resources. CD, WZ, and DL contributed in supervision, project administration, and funding acquisition. All authors read and approved the final manuscript.

## Funding

This study was supported by the National Natural Science Foundation (nos. 81830045, 81671533, 81571518, and 81971415), the National Key R&D Program of China (nos. 2016YFC1000405, 2017YFC1001402, 2018YFC1004104, and 2018YFC10029002), and the General Program of Guangdong Natural Science Foundation (No. 2020A1515010273 and 2021A1515011039). The funding bodies had no role in the design of the study, collection, analysis, or interpretation of the data in writing the manuscript.

## Conflict of Interest

The authors declare that the research was conducted in the absence of any commercial or financial relationships that could be construed as a potential conflict of interest.

## Publisher's Note

All claims expressed in this article are solely those of the authors and do not necessarily represent those of their affiliated organizations, or those of the publisher, the editors and the reviewers. Any product that may be evaluated in this article, or claim that may be made by its manufacturer, is not guaranteed or endorsed by the publisher.
