# Supplementary material for: The Risk of Postpartum Hemorrhage Following Prior Prelabor Cesarean Delivery Stratified by Abnormal Placentation: A Multicenter Historical Cohort Study
Source: Front Med (Lausanne). 2021 Oct 11;8:745080. doi: 10.3389/fmed.2021.745080 (PMC8542659; doi:10.3389/fmed.2021.745080)
Supplement: Supplementary file 1 [file Data_Sheet_1.docx]

Supplement Table 1 The demographics of the subsequent pregnancy complicating with PP and PAS

| Variables | Intrapartum CD (n=23) | Pre-labor CD  (n=340) | P |
| --- | --- | --- | --- |
| Age | 34.04±4.89 | 32.72±4.65 | 0.177 |
| BMI | 22.47±2.55 | 22.98±3.16 | 0.607 |
| Gravida | 3.48±1.24 | 3.38±1.28 | 0.594 |
| Abortion | 1.35±1.19 | 1.43±1.27 | 0.814 |
| Curettage | 1 (4.3) | 10 (2.9) | 0.518 |
| Interpregnancy interval | 93.69±48.50 | 82.69±44.12 | 0.338 |
| Weight gain | 11.58±3.69 | 12.76±3.62 | 0.063 |
| Race/ethnicity |  |  | 1 |
| Han | 23 (100) | 338 (99.4) |  |
| Others | 0 (0) | 2 (0.6) |  |
| ART | 0 (0) | 8 (2.4) | 1 |
| Source |  |  | 0.996 |
| Hospital | 13 (56.5) | 192 (56.5) |  |
| Referral | 10 (43.5) | 148 (43.5) |  |
| Mode of delivery |  |  | 1 |
| Repeat CD | 23 (100) | 339 (99.7) |  |
| TOLAC | 0 (0) | 1 (0.3) |  |
| Prom | 2 (8.7) | 14 (4.1) | 0.269 |
| Hypertension disorders | 2 (8.7) | 13 (3.8) | 0.244 |
| DM | 2 (8.7) | 55 (16.2) | 0.553 |

Data are presented as mean±SD or n (%)

CD, cesarean delivery; BMI, body mass index; ART, assisted reproductive technology; VBAC, vaginal birth after cesarean; Prom, premature rupture of membrane; DM, diabetes mellitus;

Supplement Table 2 The demographics of the subsequent pregnancy complicating with PP alone.

| Variables | Intrapartum CD  (n=22) | Pre-labor CD  (n=315) | P |
| --- | --- | --- | --- |
| Age | 34.32±5.00 | 33.73±4.59 | 0.301 |
| BMI | 21.91±2.37 | 23.01±3.33 | 0.167 |
| Gravida | 3.00±0.93 | 3.18±1.17 | 0.694 |
| Abortion | 1.09±0.92 | 1.30±1.22 | 0.623 |
| Curettage | 0 (0) | 8 (2.5) | 1 |
| Interpregnancy interval | 96.32±52.56 | 86.47±45.27 | 0.443 |
| Weight gain | 12.70±3.60 | 12.74±3.78 | 0.381 |
| Race/ethnicity |  |  | 0.591 |
| Han | 21 (95.5) | 303 (96.2) |  |
| Others | 1 (4.5) | 12 (3.8) |  |
| ART | 0 (0) | 12 (3.9) | 1 |
| Source |  |  | 0.392 |
| Hospital | 20 (90.9) | 259 (82.2) |  |
| Referral | 2 (9.1) | 56 (17.8) |  |
| Mode of delivery |  |  | 1 |
| Repeat CD | 22 (100) | 311 (98.7) |  |
| VBAC | 0 (0) | 4 (1.3) |  |
| Prom | 0 (0) | 30 (9.5) | 0.24 |
| Hypertension disorders | 0 (0) | 13 (4.1) | 1 |
| DM | 7 (31.8) | 56 (17.8) | 0.102 |

Data are presented as mean±SD or n (%)

CD, cesarean delivery; BMI, body mass index; ART, assisted reproductive technology; VBAC, vaginal birth after cesarean; Prom, premature rupture of membrane; DM, diabetes mellitus;

Supplement Table 3 The demographics of the subsequent pregnancy complicating with PAS alone.

| Variables | Intrapartum CD  (n=12) | Pre-labor CD  (n=94) | P |
| --- | --- | --- | --- |
| Age | 34.42±3.45 | 33.93±4.43 | 0.671 |
| BMI | 23.01±4.38 | 22.80±3.46 | 0.881 |
| Gravida | 2.83±1.12 | 3.54±1.49 | 0.094 |
| Abortion | 0.75±1.06 | 1.67±1.52 | 0.034 |
| Curettage | 1 (8.3) | 3 (3.2) | 0.386 |
| Interpregnancy interval | 86.25±44.63 | 93.43±44.29 | 0.582 |
| Weight gain | 12.50±2.15 | 13.93±3.73 | 0.082 |
| Race/ethnicity |  |  | 1 |
| Han | 12 (100) | 92 (97.9) |  |
| Others | 0 (0) | 2 (2.1) |  |
| ART | 0 (0) | 5 (5.4) | 1 |
| Source |  |  | 1 |
| Hospital | 10 (83.3) | 74 (78.7) |  |
| Referral | 2 (16.7) | 20 (21.3) |  |
| Mode of delivery |  |  | 0.386 |
| Repeat CD | 11 (91.7) | 91 (96.8) |  |
| VBAC | 1 (8.3) | 3 (3.2) |  |
| Prom | 3 (25) | 13 (13.8) | 0.386 |
| Hypertension disorders | 2 (16.7) | 7 (7.4) | 0.269 |
| DM | 5 (41.7) | 16 (17) | 0.058 |

Data are presented as mean±SD or n (%)

CD, cesarean delivery; BMI, body mass index; ART, assisted reproductive technology; VBAC, vaginal birth after cesarean; Prom, premature rupture of membrane; DM, diabetes mellitus;

Supplement Table 4 The demographics of the subsequent pregnancy with normal placentation.

| Variables | Intrapartum CD (n=1140) | Pre-labor CD  (n=8887) | P |
| --- | --- | --- | --- |
| Age | 33.27±4.19 | 33.06±4.37 | 0.1 |
| BMI | 22.38±3.16 | 22.52±3.24 | 0.239 |
| Gravida | 2.89±1.02 | 2.91±1.04 | 0.607 |
| Abortion | 0.90±1.02 | 0.93±1.05 | 0.376 |
| Curettage | 6 (0.5) | 96 (1.1) | 0.079 |
| Interpregnancy interval | 81.61±41.14 | 80.12±41.43 | 0.236 |
| Weight gain | 13.12±4.13 | 13.37±3.92 | 0.001 |
| Race/ethnicity |  |  | 0.091 |
| Han | 1114 (97.7) | 8745 (98.4) |  |
| Others | 26 (2.3) | 142 (1.6) |  |
| ART | 23 (2.2) | 230 (2.8) | 0.265 |
| Source |  |  | 0.483 |
| Hospital | 1030 (90.4) | 7970 (89.7) |  |
| Referral | 110 (9.6) | 917 (10.3) |  |
| Mode of delivery |  |  | 0.304 |
| Repeat CD | 1021 (89.6) | 8044 (90.5) |  |
| VBAC | 119 (10.4) | 843 (9.5) |  |
| Prom | 125 (11) | 917 (10.3) | 0.501 |
| Hypertension disorders | 42 (3.7) | 559 (6.3) | 0 |
| DM | 244 (21.4) | 1739 (19.6) | 0.143 |

Data are presented as mean±SD or n (%)

CD, cesarean delivery; BMI, body mass index; ART, assisted reproductive technology; VBAC, vaginal birth after cesarean; Prom, premature rupture of membrane; DM, diabetes mellitus;
